# Kratom (*Mitragyna speciosa*)-Induced Hepatitis

**DOI:** 10.14309/crj.0000000000000715

**Published:** 2022-04-07

**Authors:** Devin R. Allison, Muhammad Mubarak, Neal Sharma, Deepthi S. Rao

**Affiliations:** 1 Department of Pathology and Anatomical Sciences, University of Missouri, Columbia, MO; 2 Department of Medicine-Gastroenterology, University of Missouri, Columbia, MO

## Abstract

Kratom is a plant with opioid-like properties known to produce stimulant and analgesic effects. Although there are numerous studies on the psychoactive components of kratom, less is known about the toxicity. Specifically, few reports describe kratom-induced hepatotoxicity and demonstrate histological features. We provide a case report detailing the clinicopathologic findings of drug-induced liver injury caused by kratom. The laboratory workup included significant elevation of total bilirubin and alkaline phosphatase. Liver biopsy demonstrated a prominent canalicular cholestatic pattern, mixed portal inflammation, and newly described perivenular necrosis. This report provides additional information on kratom toxicity because its use continues to rise.

## INTRODUCTION

Kratom (*Mitragyna speciosa*) is a tree indigenous to Southeast Asia of which extracts from the leaves have known opioid-like properties and are marketed for treating chronic pain and for its ability to benefit those struggling with opioid addiction and withdrawal.^1–3^ Most studies of kratom focus on the psychoactive properties, whereas its toxicity is only recently gaining attention. Specifically, kratom hepatoxicity with histological examination is rarely reported but clinically significant. We report a case of kratom-associated drug-induced liver injury (DILI) to provide further insight into the symptoms and histopathology associated with its use.

## CASE REPORT

A 23-year-old man with a history of untreated cutaneous psoriasis presented with progressively worsening jaundice, diffuse itching, pale stools, dark urine, vague abdominal discomfort, mild weight loss, excessive fatigue, and easy bruising for 1 month. He had no confusion or evidence of hepatic encephalopathy. He had an exposure to hepatitis C virus 2 years before presentation. He denied any recent use of acetaminophen. He does not drink a significant amount of alcohol nor smoke cigarettes but vapes and smokes marijuana daily. He also reports that within the previous month, he ingested kratom at a high dose of 30 g per day for 14 days. His last dose was 7 days before symptom onset.

The initial laboratory tests were notable for total bilirubin of 34.3 mg/dL, alkaline phosphatase of 220 IU/L, and mildly elevated aspartate transaminase and alanine transaminase of 61 IU/L and 58 IU/L, respectively. The erythrocyte sedimentation rate was elevated at 43 mm/Hr. Ferritin was elevated at 664.0 ng/mL with a low-normal iron saturation of 20.2%. Thyroid-stimulating hormone was normal at 0.975 mcunit/mL. Coagulation studies demonstrated a mildly elevated prothrombin time of 16.2 seconds. Serum ceruloplasmin was mildly elevated at 58.4 mg/dL.

**Figure 1.**
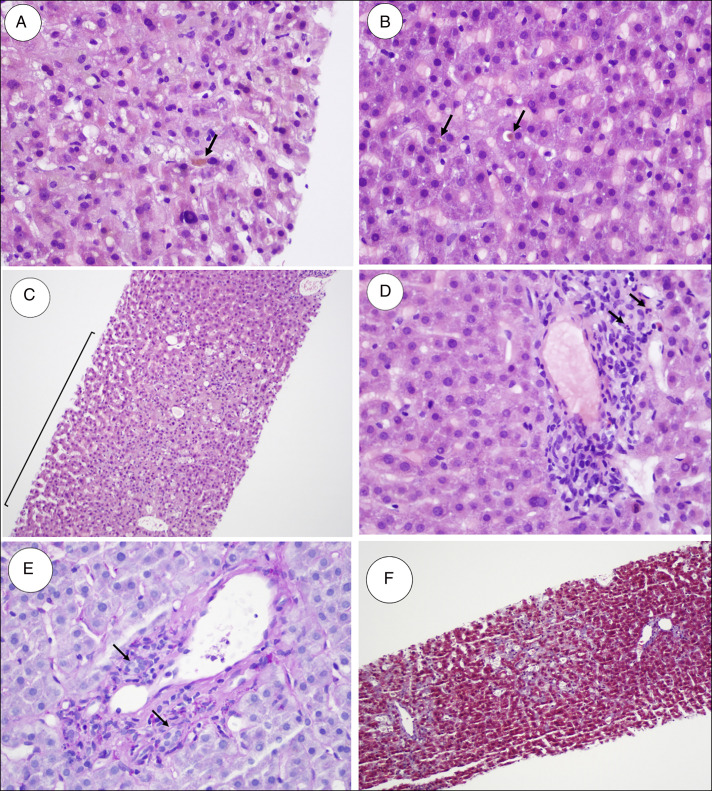
(A and B) Canalicular cholestasis in which yellow-brown chunks of concentrated bile (arrows) are present in hepatic canaliculi (hematoxylin and eosin stain, 400× magnification). (C) The bracketed area shows perivenular (acinar zone 3) necrosis which appears pale compared with the adjacent parenchyma because of the loss of hepatocytes (hematoxylin and eosin stain, 100× magnification). (D) The portal areas show minimal interface hepatitis with a mixed inflammatory infiltrate, mostly consisting of lymphocytes, but scattered neutrophils (arrows) and eosinophils are present (hematoxylin and eosin stain, 400× magnification). (E) A Periodic acid-Schiff (PAS) stain with diastase shows intact basement membranes of original bile ducts (arrows) and the associated vascular structures within the portal areas (PAS, 400× magnification). (F) A lack of periportal and perivenular fibroses, which would stain type 1 collagen bright blue if present (trichrome stain, 100× magnification). There is minimal canalicular fibrosis throughout the specimen.

Magnetic resonance cholangiopancreatography showed fatty infiltration of the liver but no structural changes, gallstones, or biliary tract abnormalities. Percutaneous ultrasound-guided random liver biopsies were obtained (Figure 1). On histopathologic evaluation, the overall architecture was mildly distorted by the presence of perivenular (acinar zone 3) necrosis noted in several foci. Sinusoidal dilatation and passive congestion were noted. The portal areas contained original bile ducts and typical vascular structures as well as a mild inflammatory infiltrate which included predominately lymphocytes with scattered neutrophils and eosinophils. The most significant finding was canalicular cholestasis which was diffusely present in the biopsy samples. No granulomas were seen. The presence of interface hepatitis was quite minimal and was not uniformly present in the biopsy. There was no bile duct damage or florid duct lesions, which was supported by a periodic acid-Schiff stain highlighting the preserved basement membranes of the original bile ducts. No periodic acid-Schiff-positive diastase-resistant cytoplasmic globules were seen. A trichrome stain on the tissue showed minimal portal and periportal fibrosis and some canalicular fibrosis. An iron stain demonstrated a minimal pathologic increase in stainable iron within hepatocytes (siderosis grade 1/4). The reticulin framework was preserved in the biopsy samples.

The initial laboratory analyses were used to generate an R ratio of 0.68, supporting a cholestatic pattern of injury (R < 2). A Roussel Uclaf Causality Assessment Method score of 6 (probable DILI) was near the maximum score of 8 for this specific scenario.^4^ The patient was diagnosed with DILI secondary to kratom and treated with ursodiol 500 mg TID, cholestyramine 4 g daily, and hydroxyzine 50 mg QID as needed. A liver transplant was considered; however, the lack of encephalopathy and preserved synthetic function of the liver rendered this a case of severe DILI and not acute liver failure. On a follow-up visit in 4 weeks, he continued to have severe pruritis and persistent jaundice, moderate abdominal pain, acholic stools, and dark urine. The laboratory workup at that time showed an even greater elevation of total bilirubin to 39.4 mg/dL and an increase of alkaline phosphatase to 333 IU/L. Oral rifampin 150 mg daily was added for pruritis. On a subsequent follow-up visit in 4 weeks, he reported improvement in his pruritus and near resolution of his jaundice. His total bilirubin had dropped to 4.63 mg/dL, and his alkaline phosphatase decreased to 156 IU/L. He did not return for further follow-up.

## DISCUSSION

Kratom extracts are commonly sold in pill or powder forms and are easily accessible in the United States and Europe through smoke shops and the internet.^2^ The herbal supplement is marketed to improve mood, relieve pain, and reduce opioid addiction by alleviating withdrawal symptoms; however, its opioid-like properties have made it a popular herb of abuse.^2,3^ The US Drug Enforcement Agency has had kratom on its “Drugs of Concern” list since 2014 and stated that more research and safety profiling must be performed before any Federal Drug Administration approved uses of kratom.^2,5^

The main active components of kratom are alkaloid compounds, including mitragynine, 7-hydroxymitragynine, and corynantheidine. Its analgesic and stimulant effects are due to their interaction with CNS µ-opioid and δ-opioid receptors and postsynaptic alpha-2 adrenergic receptors, respectively.^1–3^ In vitro cytochrome P450 inhibition studies of these alkaloid compounds demonstrated potent CYP2D6 inhibition by mitragynine and corynantheidine. CYP2D6 is known to metabolize nearly 25% of clinically used medications, which increases the concern for kratom use in those who are taking other medications or substances.^6^

Serious adverse effects of kratom include seizures, respiratory depression, and hepatoxicity.^7,8^ In cases of kratom-associated DILI, symptoms usually occur within 1–8 weeks of starting in frequent users and are characterized by fatigue, nausea, abdominal pain, dark urine, and jaundice.^9^ Although the type of injury is usually cholestatic, there are reports of mixed and hepatocellular injury. These variations may be explained by underlying liver disease or the use of concomitant medications or substances in a setting of kratom-induced alterations of metabolism.^10^

To date, there are only 5 case reports of kratom-induced liver injury that provide histological images along with detailed descriptions of the biopsy specimen (Table 1). Previously described clinical symptomatology and histology are mostly consistent with a cholestatic pattern in which canalicular cholestasis seems to be the most common histological finding.^11,12^ Three of the 4 cases also described mixed portal inflammation; however, they identified mild bile duct injury, which was not seen in our case.^13–15^ One case described an additional component of granulomatous inflammation of the bile ducts and lobular areas.^11^ We are the first to describe prominent perivenular necrosis in the setting of kratom-induced liver injury. Kratom use has been on the rise in recent decades, and it seems relevant to understand its properties and potential toxicities.^16^

**Table 1.** Cases of kratom-induced liver injury with histologic evaluation

Report	Age	Sex	Kratom use	Other substance use	Presentation	LFTs at presentation^a^				Histology	Outcome
						Total bilirubin (mg/dL)	ALT (IU/L)	AST (IU/L)	ALP (IU/L)		
Gandhi et al.	37	F	Yes	None	Nausea, decreased appetite, fatigue, jaundice, and pale stools	10.3	578	455	672	Zone 3 cholestasis, lymphocytic portal inflammation, and steatohepatitis	Improvement of symptoms and laboratory values at the 6-d follow-up.
Kapp et al.	25	M	Yes	None	Fever, chills, dark urine, abdominal pain, jaundice, and diffuse pruritis	30.9	94	66	173	Pure cholestasis	Total bilirubin 5.8 mg/dL at the 47-d follow-up.
Aldyab et al.	40	F	Yes	Nettle leaf supplements and oral contraceptive	Fever and acute abdominal pain,	5.1	875	462	162	Mixed portal inflammation, bile duct injury, granulomas, venous endotheliitis, and scattered ballooning hepatocytes	Resolution of symptoms and laboratory values at the 19-wk follow-up.
Riverso et el.	38	M	Yes	Acetaminophen	Fever, chills, dark urine, and pale stools	5.1	389	220	304	Mild mixed portal inflammation, mild bile duct injury, and mild zone 3 canalicular and hepatocellular cholestasis	Total bilirubin decrease to 1.6 mg/dL 8 d after cessation. ALT 410 and AST 142.
Fernandes et al.	52	M	Yes	Acetaminophen	Fatigue and scleral icterus	22.8	62	48	259	Marked canalicular cholestasis, mild mixed portal inflammation, mild bile duct injury, and mild lobular inflammation	Total bilirubin decrease to 4.0 mg/dL at the 4-wk follow-up.

a LFTs, liver function tests; total bilirubin (0.0–1.6 mg/dL); ALT, alanine transaminase (<50 IU/L); AST, aspartate transaminase (<40 IU/L); ALP, alkaline phosphatase (40–130 IU/L).

## DISCLOSURES

Author Contributions: D. Allison wrote the article and revised the article for intellectual content. M. Mubarak, N. Sharma, and D.S. Rao revised the article for intellectual content. D.S. Rao is the article guarantor.

Financial disclosure: None to report.

Informed consent was obtained for this case report.
